# Supplementary material for: MTrack: Automated Detection, Tracking, and Analysis of Dynamic Microtubules
Source: Sci Rep. 2019 Mar 7;9:3794. doi: 10.1038/s41598-018-37767-1 (PMC6405942; doi:10.1038/s41598-018-37767-1)
Supplement: Supplementary file 1 — Supplementary information [file 41598_2018_37767_MOESM1_ESM.pdf]

MTrack: Automated Detection, Tracking, and Analysis of Dynamic Microtubules.

Varun Kapoor[1,2,§], William G. Hirst[2,3], Christoph Hentschel[2], Stephan Preibisch[1*], Simone Reber[2,4*]

[1]Imaging Lab, Berlin Institute for Medical Systems Biology (BIMSB), Max Delbrück Center for Molecular Medicine in the Helmholtz Association (MDC), 13125 Berlin, Germany; [2]Quantitative Biology Lab, IRI Life Sciences, Humboldt-Universität zu Berlin, 10115 Berlin, Germany; [3]Research School of Biology, The Australian National University, Canberra, Australian Capital Territory 0200, Australia; [4]University of Applied Sciences Berlin, 13353 Berlin.

Supplementary Figure 1

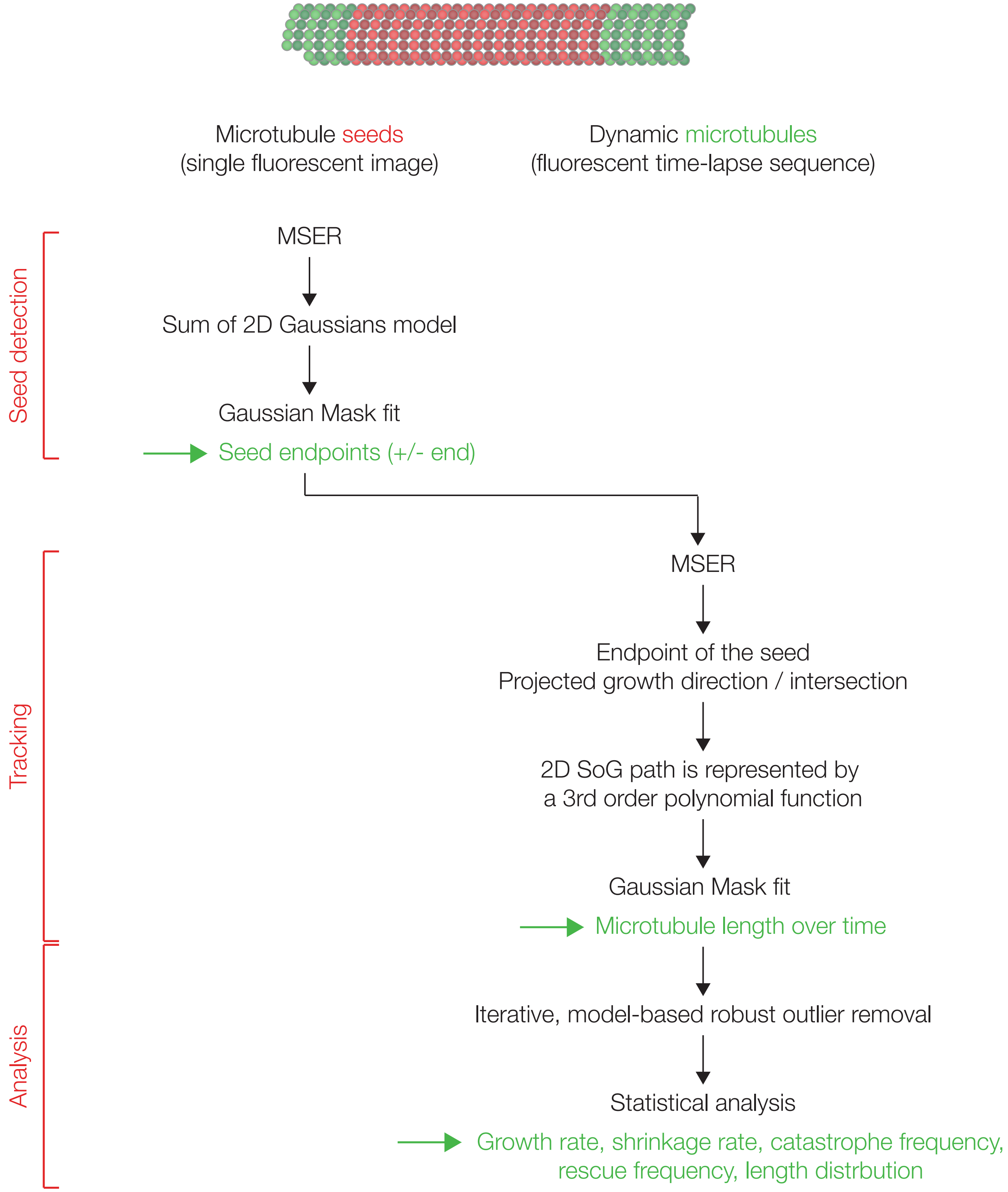

Supplementary Figure 1. Flow diagram of MTrack detecting, tracking, and analysing dynamic microtubules.

Supplementary Figure 2

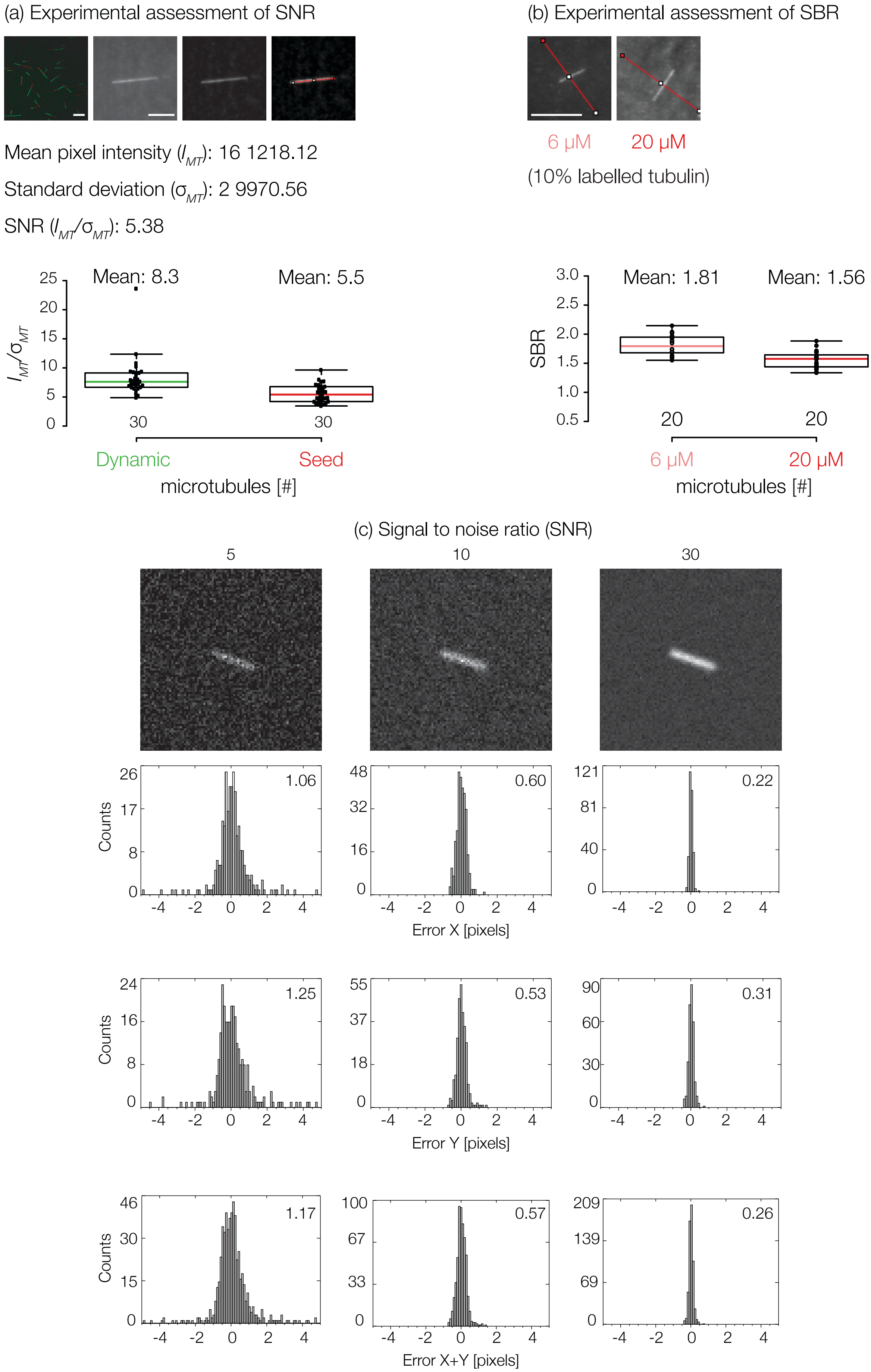

Supplementary Figure 2. Microtubule Seed Detection and Tracking Accuracy (**a**) The experimental SNRs of dynamic growing microtubules (green) and seeds (red) has been measured and calculated as described (see also Material and Methods). (**b**) Experimental assessment of the Signal-to-Background Ratio (SBR) of dynamic microtubules at 6 µM (light red) and 20 µM (dark red) total tubulin concentration. (**c**) Subpixel accuracy of simulated microtubule seed end point detection dependens on SNR. Detection error is normally distributed showing no bias towards microtubules growing in either direction. Scale bars: 10 µm.

Supplementary Figure 3

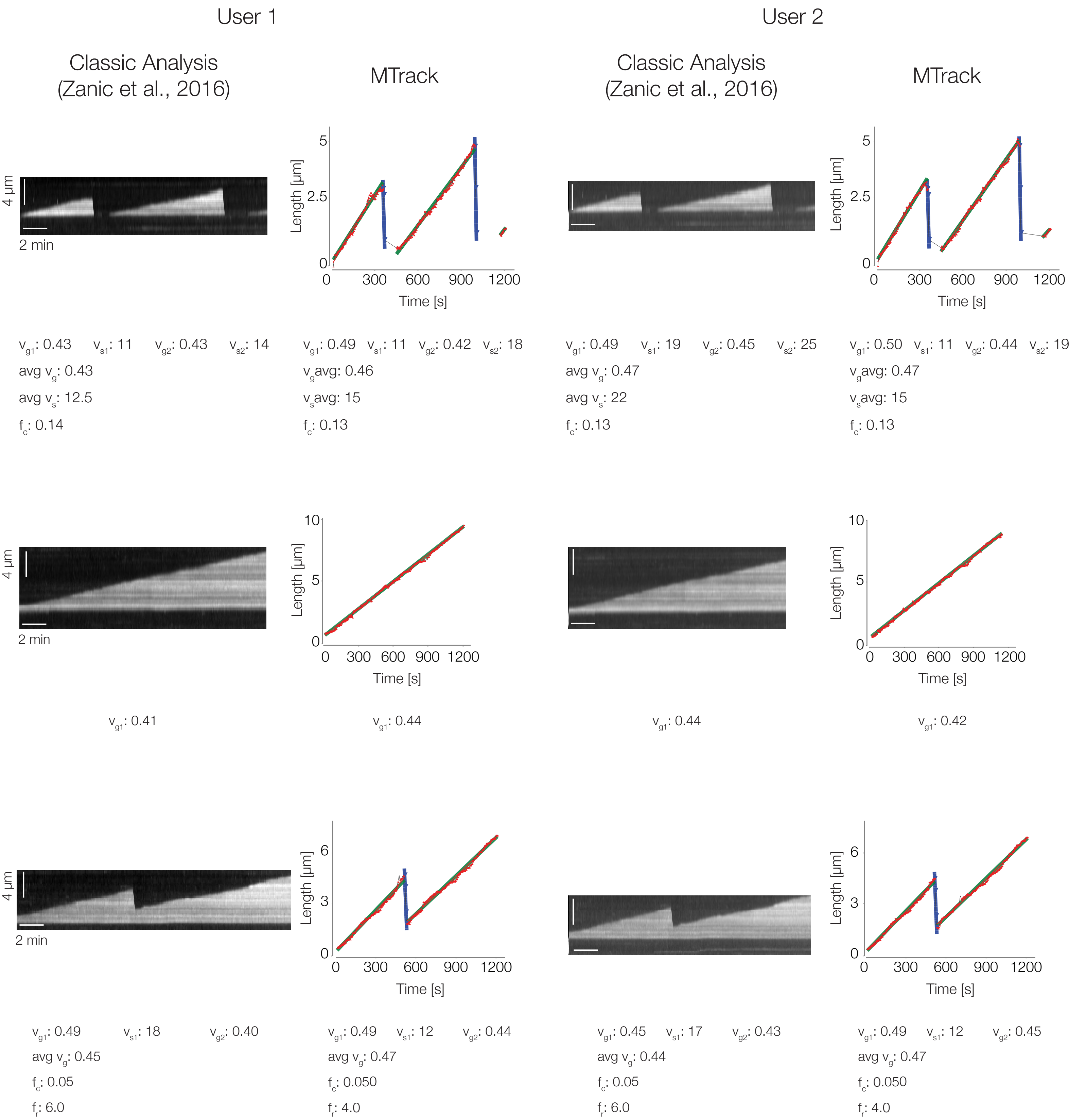

Supplementary Figure 3. Comparison between manually analyzed and computer-inferred microtubule dynamics. Polymerization velocity (v_g) and depolymerization velocity (v_s) are given in µm/min, catastrophe frequency (f_c) and rescue frequency (f_r) in s^{-1}.
